# “See one, do one, teach one”: Balancing patient care and surgical training in an emergency trauma department

**DOI:** 10.7189/jogh.12.03051

**Published:** 2022-07-06

**Authors:** Sulayman M Ayub

**Affiliations:** College of Medical and Dental Sciences, University of Birmingham, Birmingham, UK

The adage “see one, do one, teach one” refers to learning skills through a three-tiered approach as illustrated in **Figure 1**. It reflects a traditional teaching style whereby once a skill has been observed, the student/trainee is expected to perform the procedure followed by the ability to teach it. The model developed by Halsted [1] increases the responsibility of trainees. However, this concept has become less acceptable due to concerns regarding patient safety [2,3]. Technology has supported this shift in medical education; students in the UK practice procedures using a variety of simulations before meeting patients. Current developments include high-fidelity human patient simulations which provide an opportunity to refine a skill before providing care to the patient [4].

**Figure 1:**
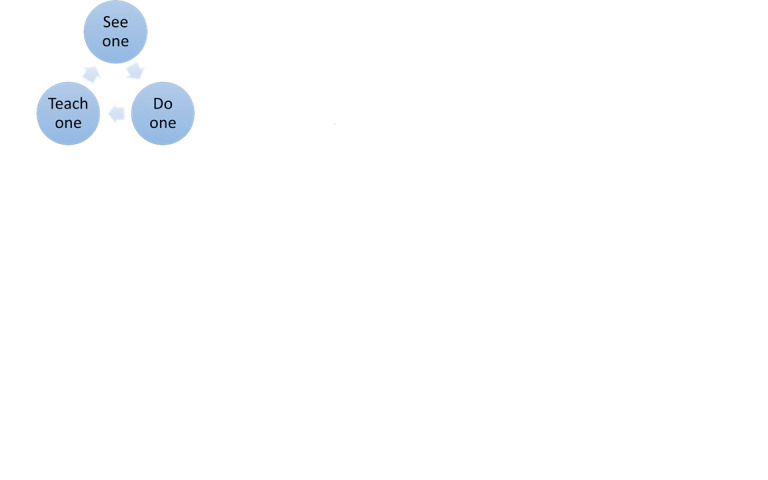
A schematic highlighting the “see one, do one, teach one” teaching method developed by Halsted [1].

Despite the advances UK students are accustomed to, international hospitals do not always have these facilities on hand. This is due to a variety of reasons including lack of resources, the need to train staff quickly, and students arriving with differing levels of competence. These apply to trauma departments in Johannesburg and result in staff relying on the aforementioned teaching style. Students conduct their electives here to gain experience in trauma-related situations and become integrated members of the surgical team. This teaching method, combined with emergency presentations, grants students a unique opportunity to get involved with a variety of procedures [5]. However, ethical issues surrounding this teaching style have been raised by the medical community advocating for patient well-being [6]. Important ethical principles must be considered and balanced fairly with the benefits this method offers surgical departments [7]. This technique should be scrutinised according to the location in which it is practised, the clinical educators that utilise it, and the students that learn from it.

**Figure:**
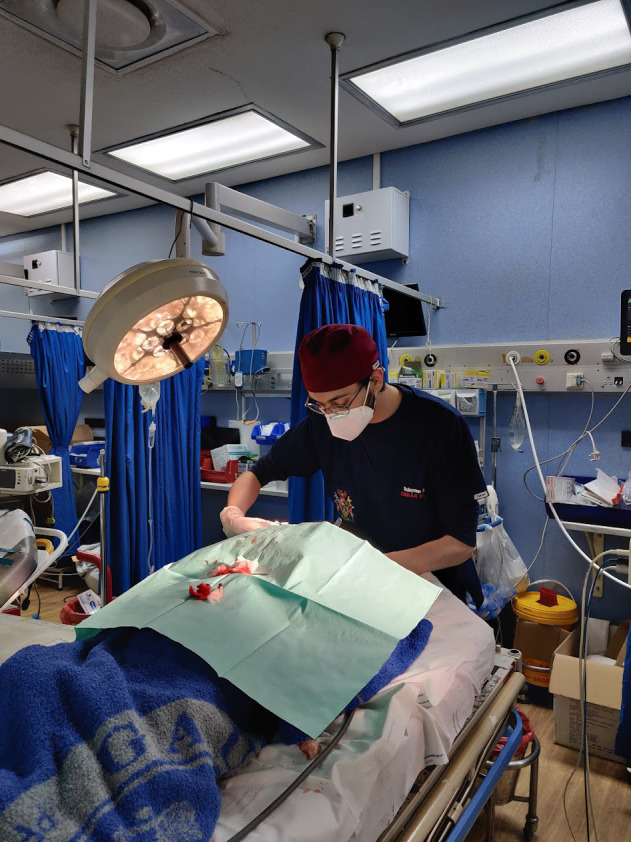
Photo: Student suturing a patient's facial wounds following competency training under the “see one, do one, teach one” teaching method. Source: Sulayman Ayub's personal collection, used with permission.

## THE SETTING

This viewpoint reflects on the teaching technique utilised in an international trauma department and identifies avenues of improvement for future elective students and elective hosts. The project was set within the emergency trauma department at Chris-Hani-Baragwanath Academic Hospital, Soweto, South Africa. As a remnant of apartheid, most of the population lives on limited resources with inadequate public infrastructure. It is the third-largest hospital in the world with 3200 beds and over 350 daily emergency cases [8]. Most of my time was spent in the emergency trauma unit where I had the opportunity to develop procedural skills such as the placement of intercostal drains and central lines. I also spent time clerking new patients, treating them if necessary, or referring them to other specialties. Due to the number of trauma cases, this department was separated from emergency medical presentations.

The doctors I worked alongside in the emergency trauma department were at varying training stages. House officers would be assigned to the trauma department as one of their early rotations and join it with minimal surgical experience. I noticed that they also partook in the aforementioned teaching method alongside students to increase their proficiency in clinical procedures. Surgical registrars leading the team in resuscitation and in the operating theatre would spend eight months in the trauma unit as part of a five-year surgical training programme; this was typically divided into two blocks of four months [8].

My reflection on the experience is divided into each individual teaching tier. The three tiers are: see one, do one and teach one.

## REFLECTIVE SUMMARY

From my first shift in the trauma department, doctors would directly say the phrase “see one, do one, teach one” and I soon realised that the doctor-student relationship within the unit was formed around this teaching culture. I was quickly exposed to a variety of procedures such as chest drains, central lines, and suturing.

### See one

This tier aims to demonstrate a procedure with a verbal explanation. Compared to book learning, visualising the process provides a clear understanding of what is expected from the surgeon [9]. Seeing a variety of procedures and management pathways increased my awareness of trauma presentations and allowed me to appreciate the need to improvise for investigations and management plans due to shortages within low-resource departments.

Throughout this tier, there are minimal ethical concerns for patient safety, albeit there are some that warrant reflection. As an observer, I had to consider a patient’s autonomy in refusing a student’s presence. Ignoring their preferences could lead to distress and uncooperativeness; these thoughts were influenced by experiencing patient-led consultations in the UK. However, I noticed a reversed dynamic between medical professionals and patients in South Africa, whereby nurses were seen as motherly figures and doctors took a more direct approach to communication. No patients refused my presence and many looked perplexed that the option was presented to them. It was equally vital that confidentiality was maintained for all patients. This was difficult due to the open plan of the emergency unit with limited rooms available for private consultations.

Furthermore, it was vital that all procedures were in the patient’s best interest and not completed for the purpose of student observation. A simple example involved a doctor asking a patient to momentarily hold his breath so I could artificially see a pneumothorax finding on an ultrasound scan. While this specific case did not impact the patient’s safety, this teaching method needs to be carefully considered.

### Do one

After observing a procedure, doctors would expect students to “do one” as the most crucial step of the teaching style. This tier should only be initiated once competency is proven through multiple observations, a clear explanation of the technique, and appropriate supervision.

This aspect of the teaching technique raises the most ethical concerns [7]. Beneficence/non-maleficence dictates that medical professionals should keep patients’ best interests at the forefront and prevent harm. During learning, all procedures must be appropriately supervised until competence is reached. If a student is dishonest regarding their capability, this can lead to serious implications for a patient’s well-being. Furthermore, it is critical that international students do not view patients as procedural practice, but that they prioritise a patient’s health as they would a family member’s. This concept links closely with patient autonomy; competent adults must be provided with adequate information about the procedure, its risks, and the person’s role in delivering it. Even if competency is equal to that of a doctor, students must be honest in their role. If a patient refuses treatment from a student, it is imperative that past practical experience is not over exaggerated to persuade them [10].

Prior to my clerkship, I was concerned about the issue of justice, and whether the need to be trained would take doctors away from what patients deserved. Doctors preferred the term “distributive justice”, whereby resource allocation is based on justified reasoning creating an equitable rather than equal system [11]. This teaching method creates trainees that enhance rather than burden the local health system; they provide more resources to be distributed within a struggling department and indirectly improve the efficiency and thus fairness of care.

### Teach one

At this stage, it is assumed the student has now amassed enough experience to perform the procedure without supervision and guide someone else through it. Teaching someone in this manner brings the learning cycle back to the start for a student who is “seeing one” for the first time. Literature and studies that call for changes to Halsted’s teaching technique often omit this tier [3,12]. Nevertheless, peer teaching is widely utilised and benefits the teachers, students, and overwhelmed doctors [13].

Teaching significantly boosts the original trainee’s confidence and provides an opportunity to recapitulate the procedure in a structured manner. I found that teaching multiple students allowed me to appreciate what others struggled with and adapt my style to support them, while simultaneously improving my own capabilities. This tier positively reinforces the need for medical professionals to become passionate educators.

However, to protect patient safety, teaching should only be initiated if the trainee is competent, able to teach effectively, and has the time to monitor new students. Furthermore, teaching procedures must be done via formal methods rather than encouraging students to develop incorrect habits.

## A MANAGEABLE SUGGESTION

Partaking in this teaching method as both a student and a teacher has given me an insight into its future use. It has contributed to the greatest lesson in responsibility within my education. This teaching method gave me the skillset, confidence and perseverance to enhance my surgical skills and support the local trauma department through lengthy shifts.

Literature expresses similar findings; valid arguments are made for this teaching approach in education because it fosters a culture of mentoring and peer-assisted learning [2]. Various teaching models support methods whereby students actively take part in the learning process [14]. This enhances their motivation to learn and encourages them to take greater responsibility for their personal development. Studies have also found that trainees taught in this manner demonstrated superior performance compared to colleagues who were trained under complete supervision [15].

Nevertheless, it is essential that patient well-being is not compromised for the purpose of surgical training and that each department openly acknowledges the ethical ramifications involved. This method also relies on an individual’s teaching technique correlating with a student’s learning style [2]. Supervisors must be willing to develop a close relationship with the student and accordingly adapt to encourage asking for assistance. Spending my time in only one hospital creates a limitation to my conclusions; other departments may not adapt to the same teaching culture or have the variety of surgical presentations required for trainees to achieve competency. Furthermore, I found that this teaching style is primarily suited to procedural skills and cannot be a substitute for learning surgical theory.

As a result, many studies call for an adaptation of the teaching method, placing a stronger emphasis on simulation and the use of procedural checklists [3,12]. This form of teaching provides an advantageous way of reducing patient safety concerns. However, low-resource trauma departments lack the infrastructure and the appropriate teaching culture required to facilitate such change. It would be preferable that a manageable recommendation be offered to such departments. From my experience, it would be beneficial for students/trainees to be provided with a structured session on the teaching method they will participate in prior to entering the emergency unit. This would encourage them to reflect upon the ethical issues involved, how to tackle them, and what steps they can take to maximise their learning potential whilst protecting patients. Further research on whether such a session changes attitudes and improves patient safety would be valuable to such departments.
